# Facile Preparation of Magnetically Separable Fe_3_O_4_/ZnO Nanocomposite with Enhanced Photocatalytic Activity for Degradation of Rhodamine B

**DOI:** 10.3390/nano14110926

**Published:** 2024-05-24

**Authors:** Li Qi, Siyu Wang, Yun Liu, Peng Zhao, Jing Tian, Baolin Zhu, Shoumin Zhang, Wenqi Xie, Huanhuan Yu

**Affiliations:** 1 College of Chemistry and Environmental Science, Shangrao Normal University, Shangrao 334001, China; seven820620@126.com (L.Q.); m18296897976@163.com (Y.L.); xwq2001@126.com (W.X.); 2 College of Chemistry, Nankai University, Tianjin 300071, China; siyu2142021@163.com (S.W.); zhaopeng@nankai.edu.cn (P.Z.); tianjingnk@nankai.edu.cn (J.T.); zhubalin@nankai.edu.cn (B.Z.); zhangsm@nankai.edu.cn (S.Z.); 3 The Key Laboratory of Advanced Energy Materials Chemistry (Ministry of Education), Nankai University, Tianjin 300071, China

**Keywords:** photocatalysis, Fe_3_O_4_/ZnO, effective interface, degradation efficiency, magnetic separation

## Abstract

Magnetic separation of photocatalysts holds great promise for water treatment. A magnetic separation method has a positive effect on the recovery of catalysts after degradation. In this paper, an efficient and reusable catalytic system is developed based on coating magnetic Fe_3_O_4_ by depositing Fe^2+^ on the surface of ZnO. The Fe_3_O_4_/ZnO nanocomposite exhibits enhanced performance for organic pollutant degradation. The Fe_3_O_4_/ZnO system demonstrates a high photocatalytic activity of 100% degradation efficiency in Rhodamine B (RhB) degradation under UV light irradiation for 50 min. The excellent photocatalytic activity is primarily due to the separation of photogenerated electron-hole pairs being facilitated by the strong interaction between Fe_3_O_4_ and ZnO. The induction of the magnetic Fe_3_O_4_ endows the Fe_3_O_4_/ZnO composite with superior magnetic separation capability from water. Experiments with different radical scavengers revealed that the hydroxyl radical (·OH) is the key reactive radical for the effective degradation of RhB. This work innovatively affords a common interfacial dopant deposition strategy for catalytic application in the degradation of organic dye pollutants and catalyst separation from wastewater efficiently.

## 1. Introduction

The rapid development of industry and agriculture has brought convenience to people, also causing serious environmental pollution, which has become one of the most important challenges that faces all living beings worldwide in the 21st century [1,2,3,4,5,6]. In particular, industrial wastewater presents a notable threat to water biodiversity and ecological balance [3]. It has been a pressing issue that needs to be addressed. Water pollution continues to be a major concern for both the ecological environment and human health [4]. The globe is facing an unprecedented energy and environmental crisis [7]. One of the main factors causing water pollution is the release of persistent organic pollutants (POPs) [5]. Among various recalcitrant organic pollutants, organic dyes possess the properties of high chroma, huge water volume, toxicity, and low biodegradability, which makes it difficult to purify dye wastewater to a suitable level before releasing it into the environment [1,8,9,10,11]. The photocatalytic oxidation method, physical coagulation method, Fenton oxidation method, and biodegradation method are now the most frequently used techniques for treating dye wastewater [2,12,13,14,15,16]. Among these treatment techniques, the semiconductor-based photocatalytic oxidation approach offers the advantages of stability, non-toxicity, high efficiency, low energy consumption, and environmentally friendly features, which has drawn a lot of interest from researchers [17,18,19,20]. Solar-driven photocatalytic oxidation is a green and efficient wastewater treatment technology [13] that can degrade toxic pollutants by decomposing organic molecules into non-toxic carbon dioxide, water, and other small molecules [6,8]. Finding efficient, inexpensive, and stable photocatalysts is one of the keys to promoting the rapid development of photocatalytic technology [9,11].

Semiconductor metal oxides occupy an important role in the photocatalytic treatment of industrial wastewater. They have the advantage of light corrosion resistance [3]. Many semiconductor metal oxides, like TiO_2_, Fe_2_O_3_, ZnO, and CuO, have been widely explored in the photocatalytic degradation process of organic dyes [12,17]. Among them, ZnO has received more attention due to its strong reducing ability and various morphologies [20]. As an efficient photocatalyst, ZnO has also demonstrated many intriguing qualities, including chemical and physical stability, strong oxidation strength, environmental friendliness, and low price [21,22,23]. However, the utilization of ZnO was not fulfilled due to its higher bandgap of ~3.3 eV [24,25], resulting in ZnO only absorbing 5% of the solar energy in UV light and exhibiting low degradation activity for organic pollutants [1], which limited its practical applications [26,27]. To overcome this drawback and improve the catalytic efficiency, an effective strategy is to utilize metal oxide particles including metal doping, constructing heterojunctions, or nanocomposites to tune the electronic structure of the host, thereby reducing the band gap [8,10,11,28,29]. Considering that semiconductor-based nanocomposite photocatalytic technologies can break down many kinds of pollutants into nontoxic molecules at room temperature and pressure economically, they are regarded as an effective technique for pollutant degradation due to synergistic effects [10,28]. In composite materials, the presence of impurities and defects caused by the dopants within the forbidden band results in a decrease in band gap energy and a lower carrier recombination rate. This allows a higher number of photogenerated electrons and holes to facilitate the decomposition of pollutant molecules, ultimately enhancing the catalytic activity of nanocomposites [28]. The other key problems with photocatalysts are recoverability and reusability, which lead to significant financial losses and pollution due to the laborious recovery process, impeding the commercialization of photocatalytic technology [30,31,32,33]. In summary, it can be seen that the high recombination rate of photogenerated carriers is the main factor limiting the performance of photocatalysts. Improving the transmission and separation efficiency of photogenerated carriers by constructing composites to enhance the photocatalytic performance as well as to improve the separation efficiency of the catalysts from water is a critical issue in the field of development of photocatalytic treatment of wastewater. In view of the above shortcomings, it is therefore an important concern to develop new and efficient photocatalysts with excellent photocatalytic activity and easy separation from water.

The application of magnetic photocatalysts in the treatment of wastewater is one of the most promising approaches to catalyst recovery [1,32,33,34]. The technique becomes more commercially feasible when an external magnetic field is utilized because it facilitates the simple separation of photocatalysts, allowing multiple recycling of the photocatalysts. As a highly magnetic material, Fe_3_O_4_ is preferred for synthesizing magnetic composites [14,33,35]. Fe_3_O_4_ has been also widely studied as a good catalyst for Fenton-like reactions [32]. Fe_3_O_4_ has the advantages of low toxicity, good stability, and good electrical conductivity [32,34]. Fe is usually considered as a suitable candidate for partial replacement in metal oxides [31]. The doping of Fe elements in catalytic systems is also an effective way to improve the efficiency of photogenerated carrier separation by modifying the band gap energy levels [29,31]. Furthermore, Fe_3_O_4_ nanoparticles could be immobilized to preserve their unique properties [32]. Stimulated by the above background, in order to inhibit the agglomeration of ZnO, adjust the band gap, improve the catalytic activity, and achieve easy separation from water, it would be an attractive strategy to fabricate a composite structure by immobilizing the magnetic Fe_3_O_4_ coating on the surface of ZnO nanoparticles. Moreover, in addition to the advantages of immobilizing magnetic nanoparticles on ZnO to improve the recovery and photocatalytic performance, the composite photocatalysts should have considerable surface area and active sites to adsorb RhB molecules, which will make it easier to degrade the pollutant molecules. To this purpose, the design of catalysts with good performance and easy separation is extremely necessary for the development of photocatalytic technology in wastewater treatment. It is important to have an understanding of the interface reconstruction and modification of composites, as well as the results of providing additional active sites, which is vital to explore the activity of photocatalytic techniques for RhB degradation.

**Scheme 1:**
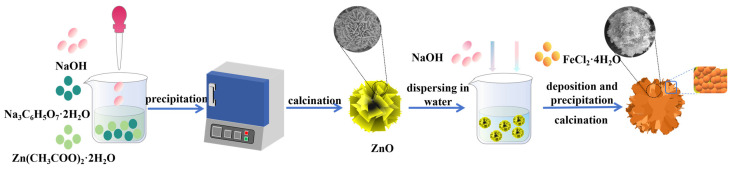
Schematic diagram for the overall preparation process of Fe_3_O_4_/ZnO.

Inspired by the above-related rationales and the significance of photocatalysis, a series of Fe_3_O_4_/ZnO composite photocatalysts were synthesized by the one-step deposition–precipitation method in this work. The synthesis process is shown in Scheme 1. Fe_3_O_4_ and ZnO can construct an effective interface by partial substitution of Zn^2+^ ions in the lattice. In this circumstance, the Fermi energy level of the Fe_3_O_4_/ZnO composite is shifted downward, which improves the catalytic activity. In addition, the coverage of Fe_3_O_4_ endows the composite with magnetic separable behavior. Furthermore, the influences of the molar ratios of Fe_3_O_4_ and ZnO, pH of the preparation process for Fe_3_O_4_/ZnO, catalyst calcination temperature, and calcination time on the photocatalytic degradation of RhB over Fe_3_O_4_/ZnO were explored. It indicates that the Fe_3_O_4_/ZnO composite has a high-temperature resistance and that surface alkalinity is beneficial for the generation of active radicals. The photocatalytic mechanism involved in the photocatalytic degradation of pollutants by Fe_3_O_4_/ZnO is also discussed. The original findings of this work provide an experiment reference for the design of highly active and easily recyclable photocatalysts by coating magnetic materials.

## 2. Materials and Methods

All chemical reagents used in this paper were of analytical grade and directly used without further purification. Zinc acetate dihydrate [Zn(CH_3_COO)_2_·2H_2_O], ferric chloride tetrahydrate [FeCl_2_·4H_2_O], and sodium hydroxide (NaOH) were purchased from Sinopharm Group Chemical Reagent Co., Ltd. (Shanghai, China). Trisodium citrate dihydrate (Na_3_C_6_H_5_O_7_·2H_2_O) was purchased from Shanghai Shenbo Chemical Co., Ltd. (Shanghai, China).

### 2.1. Synthesis of ZnO

ZnO nanoparticles were synthesized via an ordinary precipitation reaction. An aqueous solution (30 mL) with 1.76 g Zn(CH_3_COO)_2_·2H_2_O was mixed into another aqueous solution (20 mL) with 4.70 g Na_3_C_6_H_5_O_7_·2H_2_O. After being stirred at room temperature to obtain a clear solution, 0.5 mol/L NaOH was slowly added to adjust the pH to 10–11 and then the solution was stirred for three hours. After filtration, the precipitation was washed with deionized water, dried, and calcined at 500 °C for further use.

### 2.2. Synthesis of Fe_3_O_4_/ZnO

The as-synthesized ZnO was dispersed to 50 mL of deionized water and kept oscillating for 1 h. Thereafter, the FeCl_2_·4H_2_O with a Zn/Fe mole ratio of 1:1 was added and the solution was continuously oscillating for 2 h. Then, 1 mol/L NaOH was added to ensure the alkaline reaction environment (pH = 10). Then, the precipitation was filtered and washed with deionized water. At last, it was dried and calcined at 500 °C for 3 h in a muffle furnace. The final sample was ground and stored for further characterization and application. The samples with Zn/Fe mole ratios of 2:1, 3:1, and 5:1 were also prepared and denoted as Fe_3_O_4_/ZnO-2, Fe_3_O_4_/ZnO-3, and Fe_3_O_4_/ZnO-4, respectively. The effect of pH, calcination time, and temperature of Fe_3_O_4_/ZnO on the photocatalytic performance was investigated.

### 2.3. Characterization

A comprehensive range of analytical techniques was employed to characterize and investigate the physical, chemical, and optical properties of the samples. The crystal phase structure and phase composition of the samples were confirmed through powder X-ray diffraction (XRD) analysis using a MiniFlex 600 diffractometer (Rigaku, Tokyo, Japan) in the 2θ range of 10–80° with a copper target. The morphology and surface element constitution of ZnO samples calcined at different temperatures (80 °C and 500 °C) were examined using scanning electron microscopy (SEM, SU 8010, Hitachi, Tokyo, Japan) at 5 kV and transmission electron microscopy (TEM, FEI talos f200s, Thermo Fisher Scientific, Waltham, MA, USA) at specific accelerating voltages of 200 kV. Additionally, elemental mapping was performed to further analyze the samples’ properties. In the case of Fe_3_O_4_/ZnO samples, similar SEM (SIGMA 500, SIGMA Corporation, Kawasaki-shi, Japan) at 10 kV and TEM analyses were conducted, along with energy-disperse X-ray spectroscopy (EDS, XFlash 6130, Bruker, Billerica, MA, USA) and elemental mapping to study the morphology and surface element constitution. Further characterization of the samples included Fourier transform infrared spectroscopy (FTIR, Nicolet iS50 SpectrophotometerThermo Fisher Scientific) using the KBr pellet technique in the 500–4000 cm^−1^ spectral range to examine bond vibrational frequencies, UV–vis diffuse reflectance spectra (UV–vis DRS, Shimadzu UV-3600 spectrophotometer, Shimadzu, Kyoto, Japan) in the range of 200–800 nm to analyze optical properties, calculating nitrogen (N_2_) adsorption and desorption isotherms with the Brunauer–Emmett–Teller (BET) method to determine the specific surface area on Micromeritics ASAP 2460 (Norcross, GA, USA) N_2_ adsorption apparatus, photoluminescence analysis (PL, PTI, America, λ_excitation_ = 325 nm) for fluorescence properties, and electrochemical impedance spectroscopy (EIS) measurements on an electrochemical workstation (Zahner Zennium) with a standard three-electrode configuration using 0.5 M Na_2_SO_4_ as an electrolyte with a 100 kHz–0.01 Hz frequencies range for studying electrical properties. X-ray photoelectron spectroscopy (XPS) analysis was utilized to obtain the surface chemical composition of the samples. X-ray source energy is aluminum K alpha ray with a spot size of 400 µm. The binding energy calibration was performed using C 1s at 284.8 eV. The full spectrum pass energy was set at 100 eV, with a step size of 1 eV, a dwell time of 100 ms, and a single scan. For the narrow spectrum, the pass energy was 50 eV, step size 0.1 eV, dwell time 50 ms, and 5 scans were conducted. The magnetization of the nanocomposite photocatalyst was evaluated at room temperature using vibrating sample magnetometry (LakeShore 7404, LakeShore, Westerville, OH, USA) with the maximum applied field of 2.17T. The electron spin resonance (ESR) experiments were performed on a Bruker EMXplus-6/1 spectrometer. The intermediates of RhB degradation were determined by high-performance liquid chromatography-mass spectrometry (HPLC-MS, Thermo QE). As the eluent, 0.1% formic acid was used.

### 2.4. Photocatalytic Activity Measurement

RhB degradation was used to evaluate the photocatalytic properties of synthetic photocatalysts under UV light irradiation. In total, 0.015 g of sample was mixed with 60 mL of the RhB dye solution (5 mg·L^−1^) during the experiment. In order to examine the adsorption–desorption equilibrium, the suspension sample was also magnetically stirred for approximately 10 min in the dark. After that, the system was exposed to stimulated UV radiation for 50 min using a 500 W Hg lamp. Approximately 6 mL of the reaction solution suspension was collected every 10 min during this process and the deteriorated RhB solution was obtained by centrifuging the suspension. Then, using a UV–vis spectrophotometer, the maximum absorbance A at a wavelength of 554 nm was recorded to determine the concentration of RhB in the solution. The following formula was used to determine the rate of photocatalytic RhB degradation.

$$Degradation(\%)=1-\frac{C_{t}}{C_{0}}\times 100\%=1-\frac{A_{t}}{A_{0}}\times 100\%$$

where *C*_0_ and *A*_0_ represent the initial absorbance and concentration of RhB solution, respectively. The concentration and absorbance of the RhB solution in real time are denoted by *C_t_* and *A_t_*.

## 3. Results

### 3.1. Microstructure Analysis

**Figure 1:**
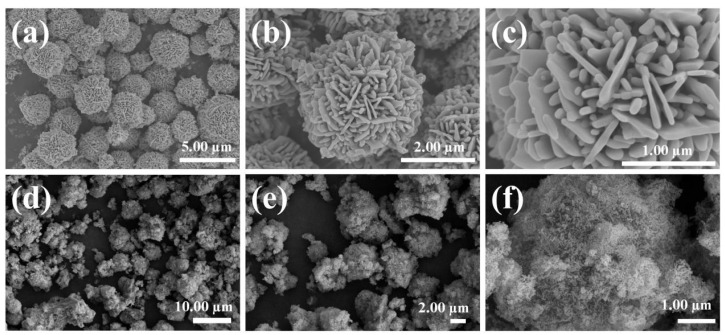
SEM images of ZnO (**a**–**c**) and Fe_3_O_4_/ZnO (**d**–**f**).

**Figure 2:**
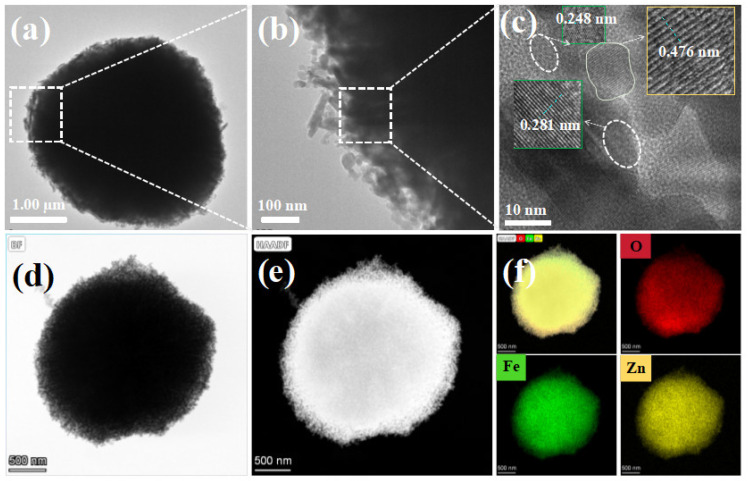
TEM image (**a**,**b**), HRTEM images (**c**), bright field TEM image (**d**), HAADF-STEM image (**e**), and element mapping images (**f**) of Fe_3_O_4_/ZnO.

SEM and TEM are utilized to analyze the morphologies of the obtained Fe_3_O_4_/ZnO. The morphology and surface micro-structure of ZnO are first magnified for comparison. The surface of ZnO displays regular shapes of hierarchical flower structure with the particle size of approximately 2 µm (Figure 1a,b), where smooth nanosheets are detected in Figure 1c. The result indicated that the hierarchical flower structure of ZnO was assembled by nanosheets. The micro morphology of ZnO is further characterized by TEM and HRTEM (Figure S1). After calcination at 500 °C, ZnO displayed a uniform hierarchical flower-like morphology (Figure S1a,b). Figure S1c shows that the lattice spacing is 0.245, which is attributed to the (101) crystal plane of ZnO (JCPDS: 36-1451) [11]. Element mapping images in Figure S1d indicates the distribution of Zn and O elements. By comparison, the SEM pictures of ZnO without calcination in Figure S2 show that the nanosheets in hierarchical flower ZnO are thinner than those in ZnO calcined at 500 °C. After coating of Fe_3_O_4_ nanoparticles, the SEM images of Fe_3_O_4_/ZnO shown in Figure 1d confirm that Fe_3_O_4_/ZnO shows a similar flower structure to pure ZnO. Irregular aggregates composed of Fe_3_O_4_ nanoparticles are observed on the nanosheet surface in ZnO (Figure 1e). Moreover, aggregates are assembled by small spherical nanoparticles, which facilitate the construction of tight interfaces in Fe_3_O_4_/ZnO, thus promoting the mass transfer of photogenerated carriers and improving the photocatalytic activity. Along with the SEM image of element distribution analysis in Figure S3, the results confirmed an even distribution of O, Fe, and Zn throughout Fe_3_O_4_/ZnO. Detailed information of the microstructure on Fe_3_O_4_/ZnO is obtained through TEM analysis (Figure 2). The TEM image in Figure 2a of Fe_3_O_4_/ZnO illustrates the stacked structure. The consistent growth of Fe_3_O_4_ nanoparticle on ZnO nanosheets is detected in the enlarged picture (Figure 2b). The HRTEM shown in Figure 2c of Fe_3_O_4_/ZnO suggests the lattice fringe spacings of 0.248 nm and 0.281 nm corresponding to the (101) and (100) crystal planes of ZnO [11,19]. In addition, a different lattice spacing of 0.476 nm is detected, which corresponds to the (111) plane of Fe_3_O_4_ (JCPDS: 19-0629) [32]. The interface is difficult to be labeled clearly in Figure 2c. It revealed the existence of a bonding interaction between Fe_3_O_4_ and ZnO [18,36]. Furthermore, the lattice spacing of ZnO in Figure S1c is mainly detected as 0.245 nm, which is notably lower than that of Fe_3_O_4_/ZnO, indicating that a few Fe ions (Fe^2+^/Fe^3+^) replaced Zn^2+^ ions in the lattice of ZnO. This confined interfacial intermediate phase could create the Zn^2+^–O–Fe^3+^ structure and induce a few effective ZnO-Fe_3_O_4_ interfaces, which is beneficial for enhancing the catalytic activity [37,38]. This result matched with SEM analysis. Furthermore, high-angle annular dark-field (HAADF) STEM images and EDS element mapping further confirm the construction of Fe_3_O_4_/ZnO composite and show even distribution of Fe element around the nanosheet ZnO (Figure 2d–f). Considering the impact of optimizing morphology through the introduction of Fe_3_O_4_, the analysis of N_2_ adsorption–desorption isotherms provides further insight into the morphological changes. The observation of type IV adsorption curves in Fe_3_O_4_/ZnO (Figure S4) suggests that the material possessed a mesoporous structure [11,12]. ZnO, Fe_3_O_4_/ZnO, and commercial Fe_3_O_4_ have BET-specific surface areas of 6.03, 32.59, and 3.91 m^2^·g^−1^, respectively. In summary, the application of Fe_3_O_4_ coating resulted in a notable enhancement of the specific surface area, hence offering a greater number of anchoring sites for mass transfer and facilitating light absorption. These analyses suggested the successful fabrication of Fe_3_O_4_ nanoparticles on the surface of the ZnO hierarchical flower.

**Figure 3:**
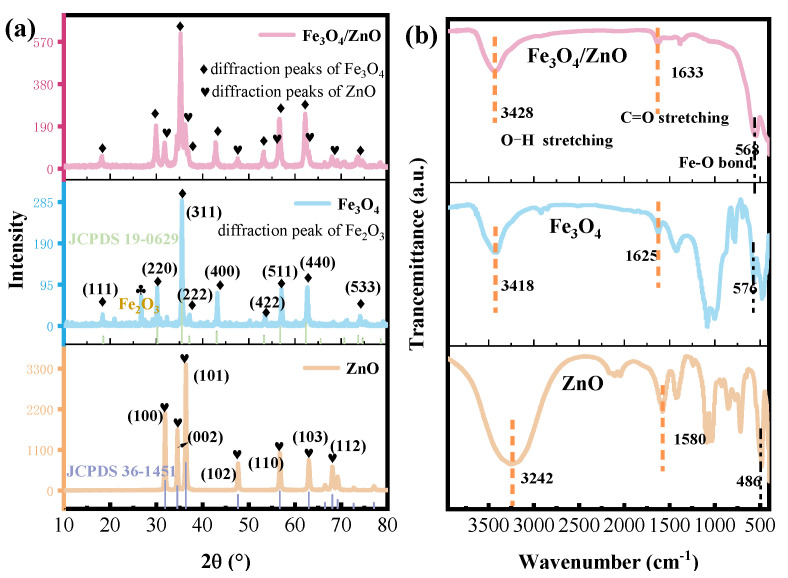
XRD patterns (**a**) and FTIR spectra (**b**) of the samples.

The material composition and crystal structure of Fe_3_O_4_/ZnO are further verified on XRD measurements. As shown in Figure 3a, for ZnO and commercial Fe_3_O_4_, the diffraction peaks are reasonably consistent with the standard card. For commercial Fe_3_O_4_, the peak at 2θ = 26.8° is ascribed to Fe_2_O_3_. The XRD pattern of Fe_3_O_4_/ZnO matches well with those of ZnO and commercial Fe_3_O_4_. The peaks observed at 2θ = 31.7°, 34.4°, 36.2°, 47.5°, 56.6°, 62.8°, and 67.9° in the Fe_3_O_4_/ZnO sample are closely ascribed to those of the ZnO phase (JCPDS: 36-1451) [6,21,35]. Additionally, the peaks recorded at 2θ = 18.2°, 30.1°, 35.4°, 37.0°, 43.1°, 56.9°, and 62.5° in the Fe_3_O_4_/ZnO sample correspond to the distinct diffraction peaks of Fe_3_O_4_ crystal planes (111), (220), (311), (222), (400), (511), and (440), consistent with pure Fe_3_O_4_ patterns (JCPDS: 19-0629) [30,32]. The results suggested the presence of pure ZnO components and validated the successful coating of Fe_3_O_4_ onto the ZnO surface. For comparison, the intensity of the diffraction peak in Fe_3_O_4_/ZnO is weaker than that in ZnO and stronger than that in commercial Fe_3_O_4_. It indicated that the unordered structure of Fe_3_O_4_ transformed to an ordered arrangement due to the interaction between Fe_3_O_4_ and ZnO. The results demonstrated the successful formation of Fe_3_O_4_/ZnO nanocomposites and particle lattice substitution by Fe ions at the interface in the Fe_3_O_4_/ZnO nanocomposites.

Additionally, the chemical composition and functional groups of the samples are further elucidated by means of FTIR. As shown in Figure 3b, Fe_3_O_4_/ZnO displays three apparent peaks. The broad absorption band in the range of 3000~3500 cm^−1^ corresponds to the O-H stretching vibrations of adsorbed water and hydroxyl groups in the samples [22,25,27]. In the 1000 to 1600 cm^−1^ region, the absorption band at ~1633 cm^−1^ is attributed to the C=O stretching vibration of carboxylic organic acids or the bending vibration of O-H bonds from H_2_O molecules [25,27,29], due to the use of zinc acetate, sodium citrate, and sodium hydroxide. It also revealed that a few hydroxide radicals were adsorbed on the surface of the composite material, which would make a contribution to high photocatalytic performance for RhB degradation by facilitating the generation of active ·OH radicals. The distinct peak at 568 cm^−1^ indicates the stretching vibrations of the Fe-O bond [30,34]. The Zn-O stretching vibration is not detected because of the relatively low crystallinity of the composite. In contrast, the hierarchical flower ZnO shows peaks at a wave number of 512 cm^−1^ assigned to Zn-O stretching vibration [22,33]. Additionally, the peaks at ~1050 cm^−1^ represent the stretching vibrations of surface C-O-C [22]. Notably, the absorption peaks of Fe_3_O_4_/ZnO at 3428 and 1633 cm^−1^ shift toward higher wavenumbers by comparison with ZnO and commercial Fe_3_O_4_, reflecting the formation of mixed metal oxide [3]. Moreover, the broadening and blue shift of the Fe-O absorption peak may be caused by the partial replacement of Zn^2+^ ions by Fe ions in the lattice. These outcomes confirmed the successful coating of Fe_3_O_4_ on the surface of ZnO, constructing the composite structure.

**Figure 4:**
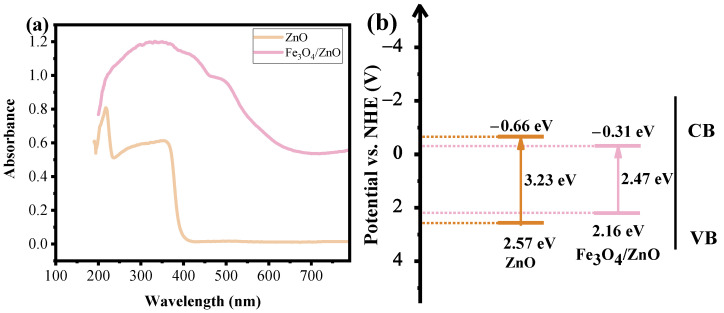
UV–vis spectra (**a**) and bandgap structure (**b**) of the samples.

The optical characterization of Fe_3_O_4_/ZnO is performed using UV–vis spectroscopy and the UV–vis spectra are illustrated in Figure 4. Figure 4a demonstrates that pure ZnO originally shows a narrow range of UV-light absorption with an edge at approximately 380 nm. The Fe_3_O_4_/ZnO composite shows a shift toward longer wavelengths in comparison to pure ZnO, with an absorption edge between 400 and 500 nm. This change in absorption edge potentially indicates the presence of Fe_3_O_4_ within the composite material and generates the interaction between Fe_3_O_4_ and ZnO, which can affect the optical properties of the ZnO [3,5]. The red shift observed may have implications for the applications of the composite material in the field of photocatalytic treatment of wastewater. The band gap energy is determined using Tauc’s equation, (αh)^2^ = A(hυ − Eg) [6,15], where n is 2 for direct transition semiconductor and n is 1/2 for indirect transition semiconductor [8,18]. α is the absorption coefficient, h is the Planck constant, υ is the frequency, and A is the absorbance constant [15,18]. By extending the line to (αh)^2^ = 0, the band gap energies of Fe_3_O_4_/ZnO and ZnO are calculated as 2.47 and 3.23 eV, respectively. Combining with valence band (VB) potentials by fitting XPS results (shown in Figure S5), the bandgap structures of the samples are inferred. The conduction band (CB) potentials are estimated using the formula E_CB_ = E_VB_ − Eg [11,19], obtaining E_CB_ values of −0.66 eV for ZnO and −0.31 eV for Fe_3_O_4_/ZnO. The potential bandgap diagrams of the samples are illustrated in Figure 4b. It can be found that the Eg of Fe_3_O_4_/ZnO is effectively decreased and the incorporation of Fe_3_O_4_/ZnO can effectively enhance light absorption, be beneficial for highly efficient charge carrier separation and transfer to generate more active free radicals during the degradation process. The results indicate that the interaction between Fe_3_O_4_ and ZnO was strengthened by the substitutional Fe doping at the interface in Fe_3_O_4_/ZnO, significantly contributing to the enhancement of photocatalytic performance.

**Figure 5:**
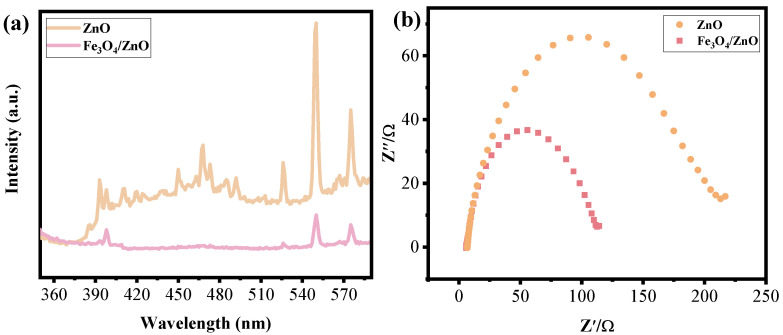
PL spectrum (**a**) and EIS Nyquist plots (**b**) of Fe_3_O_4_/ZnO and ZnO.

PL spectroscopy is used to study the recombination behavior of the photogenerated carriers. As illustrated in Figure 5a, Fe_3_O_4_/ZnO and ZnO present similar PL peaks at wavelengths of approximately 470 nm and 540 nm, respectively. The PL spectra also reveal that the quenching intensity of the PL peaks for Fe_3_O_4_/ZnO is significantly reduced compared with ZnO, suggesting remarkably suppressed recombination of photogenerated carriers [36]. The results demonstrated that the Fe_3_O_4_/ZnO composite facilitated the separation and transfer of photogenerated carriers. The separation efficiency of carriers is also evaluated using EIS. As shown in Figure 5b, Fe_3_O_4_/ZnO exhibits a smaller radius of Nyquist circle than ZnO, suggesting superior electrical conductivity and reducing the small resistance of charge transfer [19]. The results indicated that the Fe_3_O_4_/ZnO composite exhibited efficient separation and transport capabilities for photogenerated electron-hole pairs, which could help improve the photocatalytic performance for the degradation of RhB.

**Figure 6:**
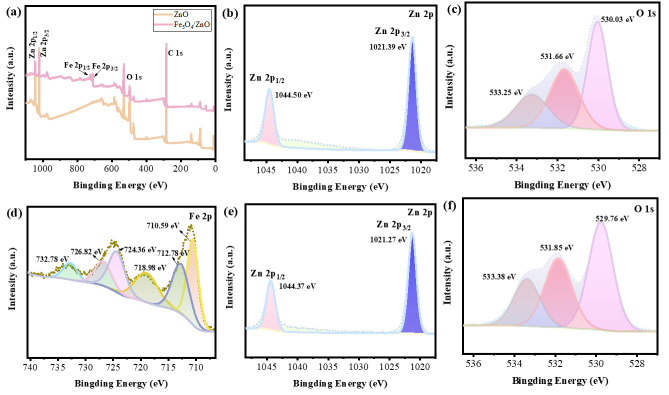
XPS survey spectra of the samples (**a**); high-resolution XPS spectra of Zn 2p (**b**) and O 1s (**c**) for ZnO; high-resolution XPS spectra of Fe 2p (**d**), Zn 2p (**e**), and O1s (**f**) for Fe_3_O_4_/ZnO.

XPS analysis is conducted to analyze the surface composition and chemical state of Fe_3_O_4_/ZnO. In Figure 6a, the survey scans XPS spectrum of ZnO shows apparent peaks at about 530 and 1022 eV, which are assigned to O 1s and Zn 2p, respectively. For Fe_3_O_4_/ZnO, new peaks located around 720 eV are detected, ascribed to Fe 2p. The results are consistent with elemental mapping analysis and EDS observation, revealing the presence of Fe, Zn, and O elements in the Fe_3_O_4_/ZnO composite due to the successful coat of Fe_3_O_4_. Figure 6b,c displays the high-resolution XPS spectra of Zn 2p and O 1s for ZnO, respectively. The Zn 2p spectrum in Figure 6b shows binding energies at 1021.39 eV and 1044.50 eV corresponding to Zn 2p_3/2_ and Zn 2p_1/2_ of Zn^2+^ [7,10,23]. The O 1s spectrum is deconvoluted into three peaks at 530.03 eV, 531.66 eV, and 533.25 eV (Figure 6c), which correspond to oxygen defects in ZnO attributing to lattice oxygen of Zn-O band, chemisorbed oxygen species, and the residual O_2_, hydroxyl (OH^−^) or water on the catalytic surface [11,19,23]. The high-resolution Fe 2p spectrum of Fe_3_O_4_/ZnO is shown in Figure 6d. It could be found that the Fe 2p spectrum can be fitted into three dominant peaks and one satellite peak. The shakeup satellite peak (718.98 eV) indicates the presence of surface Fe_3_O_4_. The peaks located at 724.36 eV and 710.59 eV, 726.82 eV and 712.78 eV should be assigned to Fe 2p_1/2_ and Fe 2p_3/2_ of Fe^2+^/Fe^3+^ species [14,35]. The broad peak at 732.78 eV is detected, which could be caused by the interaction between the Fe species and ZnO [35]. The results indicate that an effective interface between Fe_3_O_4_ and ZnO exists. Further examination of the high resolution Zn 2p and O 1s spectra of Fe_3_O_4_/ZnO show similar peaks to them of ZnO (Figure 6e,f). By comparison, the peaks of Zn 2p for Fe_3_O_4_/ZnO show about 0.12 eV blue shift to low binding energy. The electron density shift due to the dopant of Fe accounts for this negative shift phenomenon. In contrast to ZnO, the Zn atoms in Fe_3_O_4_/ZnO have a partly negative charge due to the migration of electrons to Zn caused by the presence of Fe^2+^. Additionally, owing to a partially negative charge transfer from Fe to O in the ZnO lattice, there is a negative shift in the O 1s peak for M-O, indicating a higher electron intensity [37]. These observations suggested Fe atoms were doped into the ZnO lattice. The incorporation of Fe or Zn ions at interfaces would increase the defect density and conductivity within the particles, leading to the formation of an intermediate structure [38], which confirmed the interaction between Fe_3_O_4_ and ZnO in the composites.

### 3.2. Photocatalytic Performance

#### 3.2.1. Effect of the pH Value in the Preparation of Fe_3_O_4_/ZnO

**Figure 7:**
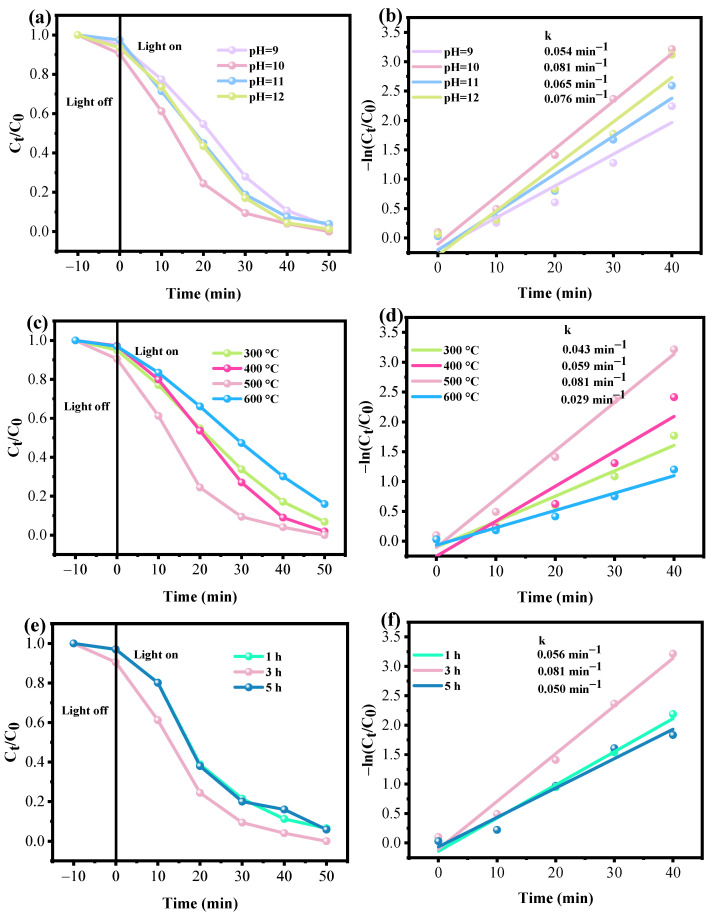
Photocatalytic activities (**a**) and first-order kinetic constants (**b**) of the degradation of RhB over Fe_3_O_4_/ZnO at different pH values of the preparation process; the photocatalytic activities of Fe_3_O_4_/ZnO annealed at different temperatures for the degradation of RhB (**c**) and the corresponding first-order kinetics constants (**d**); comparison of the evaluation of photocatalytic performance over Fe_3_O_4_/ZnO annealed at 500 °C with different calcination time (**e**); and first-order kinetic constants (**f**) of degradation of RhB.

**Figure 8:**
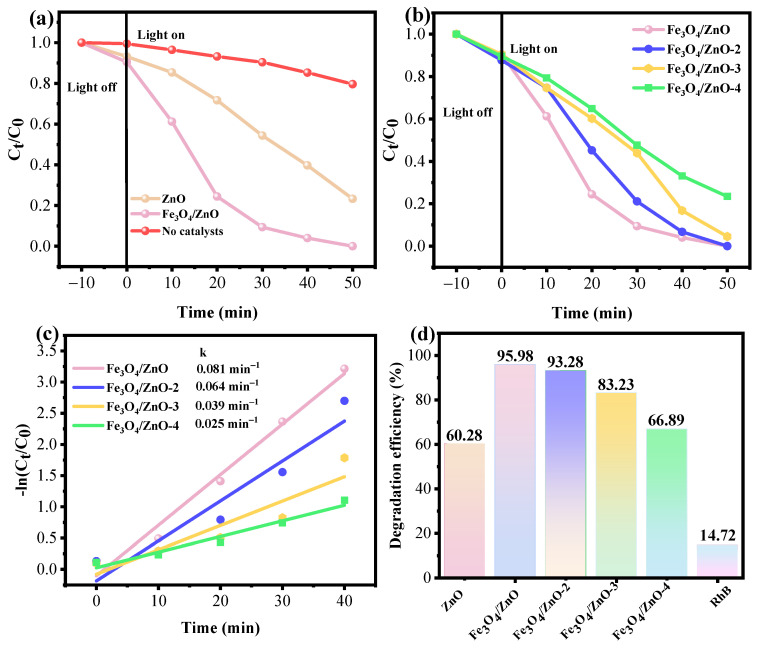
The photocatalytic degradation of RhB under simulated UV light irradiation over ZnO and Fe_3_O_4_/ZnO (**a**), comparison of evaluation of photocatalytic performance over the fabricated catalysts with a different mole ratio of Fe/Zn (**b**), the corresponding first-order kinetics constants for all the Fe_3_O_4_/ZnO cataylsts (**c**), and photocatalytic degradation efficiency of ZnO and all the Fe_3_O_4_/ZnO catalysts (**d**).

The photocatalytic degradation of RhB in an aqueous solution irradiated with UV light is used to evaluate the photocatalytic efficiency of Fe_3_O_4_/ZnO composites. The optimization procedure is conducted firstly to explore the optimal synthesis conditions of Fe_3_O_4_/ZnO nanocomposite. In the preparation process of Fe_3_O_4_/ZnO, the presence of NaOH could impact the catalytic performance by altering the quantities of active sites on the surface for the adsorption of positively charged dye molecules. The concentration of hydroxide ions (OH^−^) on the surface of Fe_3_O_4_/ZnO may lead to deprotonation of dye molecules on the catalyst’s surface, ultimately influencing physisorption by introducing weak electrostatic interactions [22]. Additionally, the OH^−^ ions may also affect the production of active radicals and influence the photocatalytic activity. To investigate the effect of surface alkalinity on the photocatalytic performance of Fe_3_O_4_/ZnO, the initial pH of the suspension was adjusted to four different values (9, 10, 11, and 12) using a 1 M NaOH solution. The molar ratio of Fe/Zn was set to 1:1. Figure 7a,b illustrates the photocatalytic performance and degradation rates for RhB removal under UV-light illumination over Fe_3_O_4_/ZnO prepared with different pH values. The degradation kinetics were described by a pseudo-first-order rate equation −ln(C_t_/C_0_) = kt [14,30], where k denotes the pseudo-first-order rate constant (min^−1^), C_0_ represents the initial RhB concentration, and C_t_ denotes the concentration at time t. Figure 8a shows that the initial pH value of the suspension does not significantly impact the absorption and degradation of RhB. When comparing the degradation rates in Figure 8b, it is clear that the highest photocatalytic degradation rate of RhB (0.081 min^−1^) occurs at an initial pH of 10. This rate is 1.5 times higher than at pH = 9. Interestingly, as the pH value increases, the degradation of RhB is not significantly changed. The k value of pH = 12 is calculated (0.076 min^−1^), which is comparable to the rate achieved with the additional NaOH adjustment to pH of 10. A possible reason for the phenomenon is given below. The highly oxidizing species, namely ·OH radicals, can be produced by the reaction between h^+^ and the chemical species OH^−^ or H_2_O [19,21]. ·OH radicals may be the active species in the photocatalytic reaction, efficiently destroying the absorbed RhB. Nonetheless, when the addition of OH^−^ in the Fe_3_O_4_/ZnO preparation process exceeds a certain value, an overabundance of OH^−^ on the surface could result in a strong electrostatic effect that would increase the electron cloud density surrounding RhB [22], which would cause RhB to desorb, additionally reducing the photocatalytic activity. Overall, the alkaline surface of Fe_3_O_4_/ZnO is conducive to the efficient degradation of RhB.

#### 3.2.2. Effect of Annealing Temperature of Fe_3_O_4_/ZnO

The influence of the annealing temperature of Fe_3_O_4_/ZnO on the photocatalytic performance, which is correlated with the crystallization, is investigated. With pH of the suspension setting at 10, Fe_3_O_4_/ZnO samples obtained at calcination temperatures of 300 °C, 400 °C, 500 °C, and 600 °C for 3 h are named as Fe_3_O_4_/ZnO-300, Fe_3_O_4_/ZnO-400, Fe_3_O_4_/ZnO, and Fe_3_O_4_/ZnO-600, respectively. In Figure 7c, it can be found that the degradation efficiency of RhB reaches 93.19%, 98.13%, 100%, and 84.06% for Fe_3_O_4_/ZnO-300, Fe_3_O_4_/ZnO-400, Fe_3_O_4_/ZnO, and Fe_3_O_4_/ZnO-600. The photocatalytic activity for RhB degradation improves gradually as the annealing temperature increases from 300 °C to 500 °C. Specifically, Fe_3_O_4_/ZnO exhibits superior photocatalytic performance, degrading 96% and 100% of RhB within 40 min and 50 min, respectively. The calculated k values of RhB for each sample are illustrated in Figure 7d. The highest k value for RhB degradation was observed in Fe_3_O_4_/ZnO, suggesting that Fe_3_O_4_/ZnO is the most effective candidate for separating photogenerated electrons and holes. This demonstrates that the degradation of RhB is greatly affected by the calcination conditions. This enhancement can be attributed to the increased crystallinity and the flower-like structure, which reduces electron-hole pair recombination sites. By comparison, the ZnO calcined at 80 °C has thinner nanosheets and besides the slight aggregation, the distribution of Zn and O elements and the morphology of the catalyst are not severely different from those of the 500 °C-calcined samples (Figure S2), suggesting that the 3D structure confers the thermal stability. With increasing calcination temperature, the flower-like structure in Fe_3_O_4_/ZnO-600 may potentially disappear. Similar phenomena has been observed in the Ce-ZnO system as well [24]. Additionally, Fe_3_O_4_ nanoparticles tend to aggregate into larger particles [32], leading to a decrease in nanoparticle diameter at higher temperatures (600 °C) and resulting in an elevated concentration of Fe_3_O_4_ units on the ZnO surface. The disruption of morphology and aggregation of Fe_3_O_4_ nanoparticles ultimately lead to a reduction in the quantity and quality of effective heterointerfaces in Fe_3_O_4_/ZnO, weakening the interaction between Fe_3_O_4_ and ZnO. As a consequence, the available number of active sites for the degradation of RhB molecules is decreased.

#### 3.2.3. Effect of Calcination Time

In order to better understand the impact of the interaction between Fe_3_O_4_ and ZnO on the photocatalytic activity of RhB degradation, the effect of calcination time is investigated in the Fe_3_O_4_/ZnO system. The nano-composite photocatalysts calcined at 500 °C for 1 h, 3 h, and 5 h are denoted as Fe_3_O_4_/ZnO-1h, Fe_3_O_4_/ZnO, and Fe_3_O_4_/ZnO-5h, respectively. In Figure 7e, after irradiation for 30 min, about 79%, 90%, and 80% of RhB is decomposed for Fe_3_O_4_/ZnO-1h, Fe_3_O_4_/ZnO, and Fe_3_O_4_/ZnO-5h, respectively. In Figure 7f, Fe_3_O_4_/ZnO presents high photocatalytic activity with the first order kinetic constant of 0.081 min^−1^, which is 1.45 times and 1.62 times to Fe_3_O_4_/ZnO-1h and Fe_3_O_4_/ZnO-5h. It is evident that the effect of calcination time on the activity is not obvious but there is a risk of agglomeration of the samples with the extension of calcination time, which leads to the decrease in activity. These findings supported the notion that the effective interaction between Fe_3_O_4_ and ZnO could significantly impact the photocatalytic performance of the Fe_3_O_4_/ZnO nanocomposite photocatalysts.

#### 3.2.4. Photocatalytic Activity of Fe_3_O_4_/ZnO

The impact of the Zn/Fe mole ratio on the performance of Fe_3_O_4_/ZnO is studied to identify the optimal conditions for synthesizing Fe_3_O_4_/ZnO. The samples with the Zn/Fe mole ratio of 1:1, 3:1, 4:1, and 5:1 are named as Fe_3_O_4_/ZnO, Fe_3_O_4_/ZnO-2, Fe_3_O_4_/ZnO-3, and Fe_3_O_4_/ZnO-4, respectively. Figure 8a illustrates that about 14.72% of RhB could be degraded by UV light in the absence of a catalyst. This was driven by the self-photosensitization of the RhB molecule. When exposed to UV light, the Fe_3_O_4_/ZnO composite exhibits superior photocatalytic activity in RhB degradation compared to pure ZnO. This is attributed to the enhanced photocatalytic degradation rate resulting from the interaction between Fe_3_O_4_ and ZnO, which effectively inhibits the recombination of photogenerated carriers, in turn, facilitating sufficient light harvesting and catalytic activity. However, the strong magnetic properties of commercial Fe_3_O_4_ cause it to adsorb onto the surface of the rotor during catalytic reactions, preventing it from playing a catalytic role. Therefore, commercial Fe_3_O_4_ is not utilized in control experiments. Figure 8b depicts that Fe_3_O_4_/ZnO exhibits the highest photocatalytic activity for RhB degradation, achieving approximately 90% degradation efficiency within 30 min of UV light irradiation. Conversely, Fe_3_O_4_/ZnO-2, Fe_3_O_4_/ZnO-3, Fe_3_O_4_/ZnO-4, and ZnO only achieved about 79%, 56%, 52%, and 46% degradation efficiency, respectively. The addition of higher amounts of Fe_3_O_4_ results in a slight decrease in photocatalytic activity. This can be attributed to the reduced separation efficiency of photogenerated carriers caused by the abundance of Fe_3_O_4_ covering the surface of ZnO in the state of aggregation leading to heterointerfaces being weakened. Thus, the carrier transfer is impeded and the quantity and quality for the exposure of active sites also decrease. Consequently, this attenuation in photogenerated carrier separation negatively impacts the photocatalytic performance of the composite photocatalysts. Similar findings have been reported in other nanocomposite photocatalysts such as CuInS_2_/ZnO [7] and NiS/ZnO [18]. These findings implied that the optimal mole ratio of Fe_3_O_4_ and ZnO was 1:1 in the Fe_3_O_4_/ZnO composite for improving photocatalytic activity. Taking into consideration the above analysis, the optimum synthesis conditions of Fe_3_O_4_/ZnO composite were pH of 10, calcination temperature of 500 °C, calcination time of 3 h, and Zn/Fe mole ratio of 1:1. The sample synthesized under these conditions is named as Fe_3_O_4_/ZnO.

Figure 8c demonstrates rate constants, k, of 0.081 min^−1^, 0.064 min^−1^, 0.039 min^−1^, and 0.025 min^−1^ for Fe_3_O_4_/ZnO, Fe_3_O_4_/ZnO-2, Fe_3_O_4_/ZnO-3, and Fe_3_O_4_/ZnO-4, respectively, indicating that the photocatalytic degradation rate of Fe_3_O_4_/ZnO is the fastest under UV light irradiation. Fe_3_O_4_/ZnO shows a significantly high photocatalytic degradation rate, which is approximately 3.85 times that of ZnO (Figure S6). Figure 8d illustrates the degradation efficiency of various samples after 40 min of irradiation. It is evident that Fe_3_O_4_/ZnO achieved the highest degradation efficiency, with approximately 96% of RhB being degraded. These findings indicate that Fe_3_O_4_/ZnO possesses the capability of modeling photogenerated electron-hole separations. The incorporation of Fe into the ZnO lattice plays a crucial role in enhancing the interaction between Fe_3_O_4_ and ZnO. On one hand, Fe^3+^ acts as an electron trap, facilitating the separation of photogenerated electrons and holes. On the other hand, the presence of Fe increases internal defects in ZnO, serving as recombination centers for photogenerated charge carriers. With optimal Fe doping content, the formation of heterojunctions at the interfaces between Fe_3_O_4_ and ZnO facilitates the separation of photogenerated electron-hole pairs, resulting in enhanced photocatalytic efficiency. These results are in accordance with HRTEM, FTIR, and XPS analyses. To demonstrate the good degradation of Fe_3_O_4_/ZnO with the interaction between Fe_3_O_4_ and ZnO, the photocatalytic degradation efficiencies are also compared with other similar studies (Table S1) and the results showed that Fe_3_O_4_/ZnO had good photocatalytic RhB degradation activity.

### 3.3. Cycle and Magnetic Separation Performances

**Figure 9:**
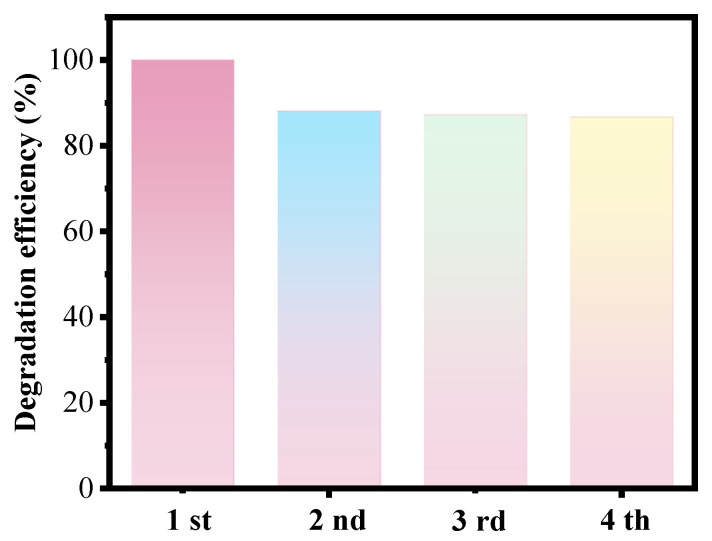
Photocatalytic RhB degradation efficiency of cycling tests over Fe_3_O_4_/ZnO.

**Figure 10:**
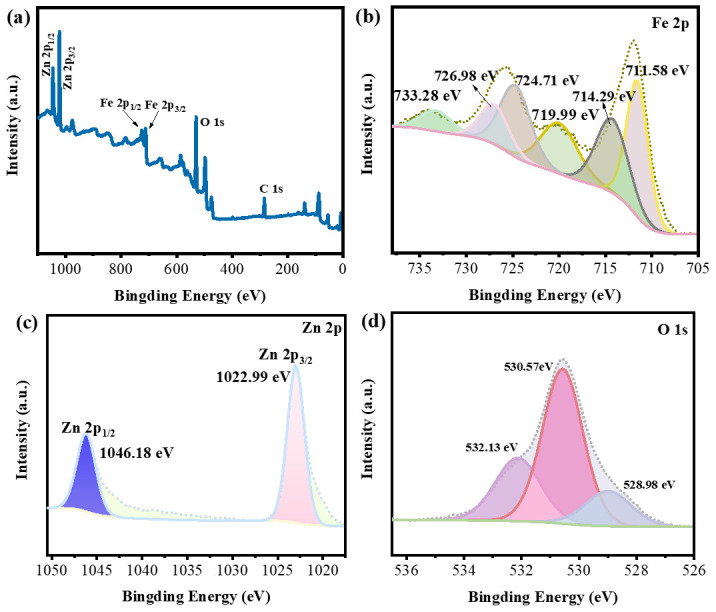
XPS spectra of reused Fe_3_O_4_/ZnO nanocomposite. Survey scan (**a**), Zn 2p (**b**), Zn 2p (**c**), and O 1s (**d**).

The recycling and magnetic separation performances of Fe_3_O_4_/ZnO for photocatalytic degradation of RhB are tested to evaluate the practical reusability and recyclability of the nanocomposite photocatalyst. The results depicted in Figure 9 indicate that a similar level of RhB photodegradation efficiencies is maintained after four cycles, indicating that the Fe_3_O_4_/ZnO catalyst has good recyclability. This suggests that the nanocomposite photocatalyst shows promise for long-term use in the degradation of RhB, showcasing its potential as a sustainable and effective solution for wastewater treatment. The slight decrease in the degradation rate may be due to the loss of catalyst during the photocatalytic cyclic experiments or the partial overflow of Fe atoms to the surface of the nanocomposite. Meanwhile, in order to verify the stability of the catalyst, XPS tests were also performed on the reused catalysts. As can be seen from Figure 10a, The full-spectrum scanning results show that Fe, Zn, and O elements were present in the sample, which was consistent with XPS results of the fresh Fe_3_O_4_/ZnO catalyst. As can be seen from the Fe2p XPS spectrum (Figure 10b), the presence of Fe^3+^ ions and Fe^2+^ in the Fe_3_O_4_/ZnO composite is evidenced by the fitted peaks at 711.58 eV, 714.29 eV (Fe 2p_3/2_), 724.71 eV, 726.98 (Fe2p_1/2_), and 718.98 eV. As shown in Figure 10c, the high-resolution XPS spectrum of Zn 2p showed two peaks at 1046.18 and 1022.99 eV belonging to the Zn 2p_1/2_ and Zn 2p_3/2_ orbitals of ZnO, indicating the presence of Zn^2+^ in the composite [7]. In the O 1 s spectrum (Figure 10d), there are also three peaks at 532.13 eV, 530.57 eV, and 528.98 eV, which are related to ZnO and Fe_3_O_4_, respectively [8,14,24,30]. By comparing the data to the fresh samples, it could be found there were obvious chemical and structure changes in the Fe_3_O_4_/ZnO composite due to the overflow of Fe atoms from the lattice to the surface, which would decrease the catalytic activity of Fe_3_O_4_/ZnO. Thus, it could be concluded that the slight decrease in RhB degradation efficiency after four cycling tests is attributed to the partial migration of Fe ions. The magnetic properties of both commercial Fe_3_O_4_ nanoparticles and the Fe_3_O_4_/ZnO nanocomposite are evaluated at room temperature, revealing differences in saturated magnetizations (Figure S7). It could be found that the commercial Fe_3_O_4_ nanoparticles have a saturated magnetization of 69.22 emu/g, while the Fe_3_O_4_/ZnO nanocomposite has a lower value of 3.68 emu/g due to the presence of nonmagnetic ZnO. However, despite the reduction in magnetization, the nanocomposite still maintained a sufficiently high level of magnetization to allow the separation by an external magnetic field (inset in Figure S7). This characteristic would facilitate the separation of photocatalysts from treated solutions, making the nanocomposite a promising material for environmental remediation purposes. Overall, the stability and the effectiveness of magnetic separation boost the potential applications of the Fe_3_O_4_/ZnO nanocomposite in meeting environmental challenges.

### 3.4. Photocatalysis Mechanism of Fe_3_O_4_/ZnO

**Figure 11:**
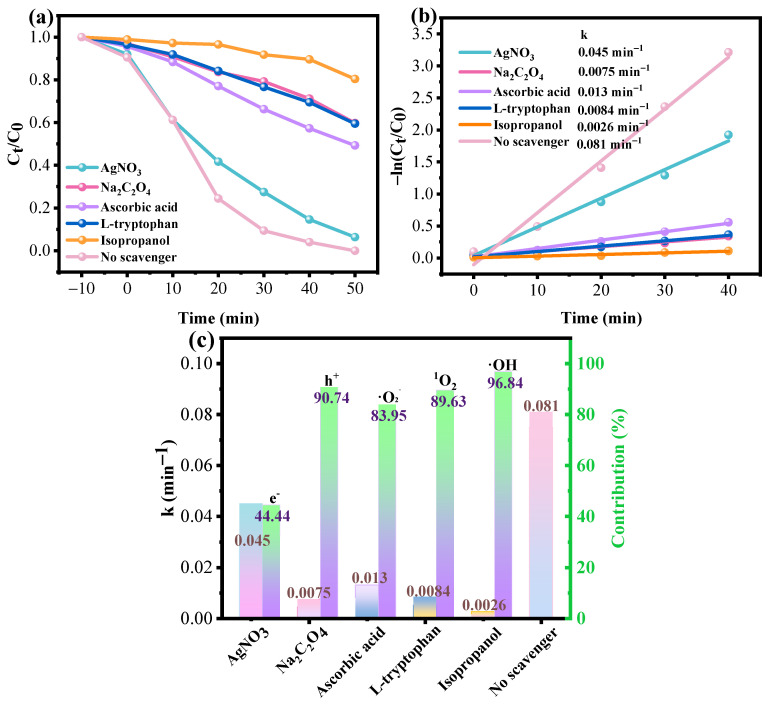
Effect of active species scavengers (**a**), pseudo-first order kinetic curves (**b**), and active species contribution percentage (**c**) on the degradation of RhB, using Fe_3_O_4_/ZnO photocatalyst under UV light irradiation.

To determine the primary active species responsible for the photodegradation of RhB in this research, various sacrificial agents (AgNO_3_, Na_2_C_2_O_4_, ascorbic acid, L-tryptophan, and isopropanol) are utilized to scavenge photogenerated electrons (e^−^), holes (h^+^), superoxide radicals (·O_2_^−^), singlet oxygen (^1^O_2_), and hydroxyl radicals (·OH) [19,31]. Figure 11a illustrates the photocatalytic performance of Fe_3_O_4_/ZnO in the presence of a sacrificial agent. After the addition of sacrificial agents, the degradation efficiency of RhB decreases. The degradation rate decreases from 0.081 min^−1^ (without scavenger) to 0.045 min^−1^ (AgNO_3_), 0.0075 min^−1^ (Na_2_C_2_O_4_), 0.013 min^−1^ (ascorbic acid), 0.0084 min^−1^ (L-tryptophan), and 0.0026 min^−1^ (isopropanol), respectively (Figure 11b). The percentage contributions of active radicals calculated by using the equation ($\frac{k-k_{radical}}{k}$ × 100%), as shown in Figure 11c, reveal that isopropanol, L-tryptophan, Na_2_C_2_O_4_, and ascorbic acid significantly inhibit the photocatalytic degradation of RhB, while the impact of AgNO_3_ is less pronounced. The photocatalytic degradation rate of RhB decreases the greatest with the addition of isopropanol, indicating the dominant role of hydroxyl radicals (·OH) in the photocatalytic process. Based on these findings, it can be inferred that the order for the contribution of all the radicals in the photocatalytic degradation of RhB was ·OH > h^+^ > ^1^O_2_ > ·O_2_^−^ > e^−^. These results suggest that ·OH, h^+^, ^1^O_2_, and ·O_2_^−^ are involved in RhB degradation, among which ·OH is the major radical. The contribution of photogenerated e^−^ is minimal. This is because Fe^3+^ ions acting as capture sites could trap electrons and then be reduced to Fe^2+^. Fe^2+^ ions would react with O_2_ to generate ·O_2_^−^ and Fe^3+^ ions [27,34].

**Figure 12:**
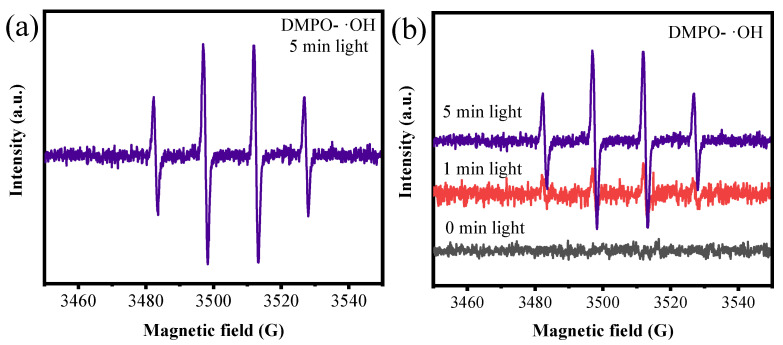
ESR spectra of Fe_3_O_4_/ZnO for DMPO-·OH under UV light irradiation for 5 min (**a**) and at different time intervals (**b**).

In addition, ESR experiments are carried to determine the presence of ·OH species in the photocatalytic RhB degradation process. The ESR spectra were obtained using DMPO as a trapping agent for ·OH [9]. As shown in Figure 12a, the ESR spectrum showed the signals of ·OH radical after UV irradiation exposure (5 min). As shown in Figure 12b, the signal peak intensity was enhanced with the increase in UV light exposure time. And no radical generation was observed under dark conditions. The above results indicated that ·OH could be generated during the catalytic reaction, which was consistent with the results of radical trapping experiments. This mechanism is supported by the following chemical equation:

Fe_3_O_4_/ZnO + UV light → Fe_3_O_4_/ZnO (e^−^) + Fe_3_O_4_/ZnO (h^+^) (1)

h^+^ + H_2_O/OH^−^ → ·OH (2)

Fe^3+^ + e^−^ → Fe^2+^ (3)

Fe^2+^ + O_2_ → ·O_2_^−^ + Fe^3+^ (4)

e^−^ + O_2_ → ·O_2_^−^ (5)

UV light + O_2_ → ^1^O_2_ (6)

h^+^ + ·O_2_^−^ → ^1^O_2_ (7)

RhB + ·OH/h^+^/^1^O_2_/·O_2_^−^ → CO_2_ + H_2_O + products (8)

When exposed to UV light, the electrons (e^−^) in the VB of ZnO are stimulated and moved to CB, creating holes (h^+^) in the VB (Equation (1)). Following this, h^+^ remaining in the VB of ZnO interacts with H_2_O or OH^−^ molecules on the catalyst surface, resulting in the formation of ·OH radicals (Equation (2)). Meanwhile, e^−^ remaining in the CB reacts with Fe^3+^, resulting in the conversion of Fe^3+^ to Fe^2+^ (Equations (2) and (3)). Fe^2+^ or e^−^ reacts with the O_2_ molecules absorbed on the catalyst surface, leading to the creation of ·O_2_^−^ radicals (Equations (4) and (5)). Based on the pathway through which ^1^O_2_ is produced, the O_2_ molecules could be excited by UV light to form ^1^O_2_ (Equation (6)). ^1^O_2_ could also be generated through the combination of h^+^ and ·O_2_^−^ radicals (Equation (7)). The ·OH, h^+^, ^1^O_2_, and ·O_2_^−^ radicals exhibit strong oxidative properties, breaking down organic RhB pollutants into harmless small molecules (Equation (8)).

**Figure 13:**
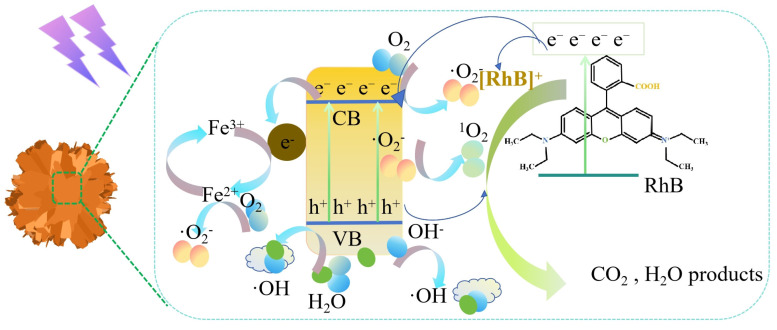
Possible photocatalytic mechanism in the Fe_3_O_4_/ZnO system.

A possible mechanism for the photocatalytic degradation of RhB over Fe_3_O_4_/ZnO is depicted in Figure 13 based on the experimental results and conclusions presented earlier. The process involves the generated electron-hole pairs upon light irradiation, which leads to the production of active oxygen species. These active species then react with RhB molecules, breaking them down into smaller less harmful compounds. Initially, the generation of e^−^ and h^+^ pairs occurs within ZnO and e^−^ transitions from VB to CB. This process leads to the creation of free-charge carriers that can participate in photocatalytic reactions. Subsequently, h^+^ in the VB can partake in oxidation reactions. For example, h^+^ reacts with H_2_O or OH^−^ in the system to produce ·OH. Additionally, e^−^ in the CB is mobile and has the potential to participate in reduction reactions. Thus, ·O_2_^−^ can be obtained by the reaction of O_2_ and e^−^ in CB or in the conversion process of Fe^3+^ to Fe^2+^. As a reductant, Fe^2+^ reacts with O_2_ to form ·O_2_^−^ radicals. ·O_2_^−^ or O_2_ can also serve as reactants to produce ^1^O_2_, which also aids in the oxidation of organic pollutants. The organic dyes absorbed on the surface of Fe_3_O_4_/ZnO can be broken down and degraded by the active radicals. The degradation of RhB is predominantly driven by ·OH radicals. Considering the photosensitivity, the photocatalytic mechanism degradation of RhB over Fe_3_O_4_/ZnO photocatalyst should also involve the self-photosensitization-driven degradation of the RhB molecule, which was proven in Figure 8. In this pathway, when irradiated under UV light, the RhB molecule becomes excited, causing the e^−^ to be injected into the CB of ZnO. The injected e^−^ then interacted with the adsorbed catalysts or O_2_ molecules, resulting in the formation of active species. This led to the mineralization of RhB and the creation of the final product. The photocatalytic degradation process of RhB demonstrates that Fe_3_O_4_/ZnO is an effective photocatalyst for the degradation of organic pollutants in water.

In addition, the primary intermediates in the Fe_3_O_4_/ZnO photocatalysis system after 20 min of photocatalytic degradation are detected and characterized using HPLC-MS. The possible degradation pathways of RhB are proposed in Figure S8. By comparing the results with those from previous studies and combining them with the findings from HPLC-MS analysis [5,13], along with the catalytic mechanism outlined above, the main possible degradation of RhB is presented, which should involve the successive loss of the bis(diethylamino) substituent alkyl group by the attack of active radicals from the molecular structure. The mass spectra of the main intermediates are shown in Figure S9. The first degradation pathway starts with the breaking of the C*_sp_*_3_–N bond, resulting in the removal of the ethyl group and the formation of a product with P2 (*m*/*z* = 415). Subsequent oxidations lead to the removal of more ethyl groups, resulting in products with P3 (*m*/*z* 387), P4 (*m*/*z* 359), and finally P5 (*m*/*z* 331). Further oxidative ring-opening processes result in the product with P6 (*m*/*z* 310). The second degradation pathway involves the oxidative hydroxylation of RhB, leading to the formation of a product with P7 (*m*/*z* 459). This product undergoes further oxidation and de-ethylation steps, resulting in products with P8 (*m*/*z* 431), P9 (*m*/*z* 336), P10 (*m*/*z* 338), and P6 (*m*/*z* 310). Additionally, the oxidative cleavage of the ring leads to the formation of a product with P11 (*m*/*z* 477), which is further oxidized to P12 (*m*/*z* 436) by removing the dimethylamine group and then degraded to P13 (*m*/*z* 408) through de-ethylation. These degradation processes ultimately yield small molecule acids, amines, and carboxylic acid compounds like HCOOH and CH_3_COOH. These compounds continue to be oxidized to form small molecules like H_2_O, CO_2_, or other products.

## 4. Conclusions

The magnetic nanocomposite Fe_3_O_4_/ZnO photocatalyst is prepared by a deposition–precipitation process, which is utilized in degrading organic pollutants. The Fe_3_O_4_/ZnO nanocomposite performs good activity for photocatalytic degradation of RhB with 100% degradation efficiency after irradiation of 50 min. The key active radical is ·OH. The improved photocatalytic activity under UV light is attributed to the enhanced separation of photo-induced carriers deriving from the interfacial effect of the strong interaction between Fe_3_O_4_ and ZnO. The 3D flower-like structure with high stability of Fe_3_O_4_/ZnO nanocomposite provides fixed sites for the deposition of Fe_3_O_4_ and creates effective heterointerfaces. The Fe_3_O_4_/ZnO catalyst also preserves good stability and magnetic separation properties. In summary, the facile synthesis process provides a convenient approach for the preparation of magnetic photocatalysts. Fe_3_O_4_/ZnO demonstrates significant potential for applications in environmental remediation and water treatment.

## Data Availability

Data are contained within the article and Supplementary Materials.

## Supplementary Materials

The following supporting information can be downloaded at https://www.mdpi.com/article/10.3390/nano14110926/s1. Figure S1: TEM image (a), HRTEM image (b), enlarged HRTEM image (c), and elemental mapping for O and Zn (d–f) of ZnO calcined at 500 °C; Figure S2: SEM images (a–c), elemental mapping for O and Zn (d–f) of ZnO dried at 80 °C; Figure S3: SEM image (a) with EDS image (b) and elemental mapping for O, Fe, and Zn (c–f) of Fe_3_O_4_/ZnO; Figure S4: The N_2_ adsorption–desorption isotherms of ZnO, Fe_3_O_4_/ZnO, and commercial Fe_3_O_4_; Figure S5: VB-XPS spectra of ZnO (a) and Fe_3_O_4_/ZnO (b); Figure S6: The corresponding first-order kinetics constants for the ZnO catalyst; Figure S7: Magnetization curves for Fe_3_O_4_/ZnO nanocomposite (a) and commercial Fe_3_O_4_ nanoparticles (b). Inset figure in (a) shows the separation process of Fe_3_O_4_/ZnO from the degraded RhB solution by using a magnet; Figure S8: Possible degradation pathways of RhB. Figure S9: Mass spectra of main intermediates of RhB solutions after irradiation for 20 min under simulated UV light. Table S1: Comparisons of RhB photocatalytic degradation between Fe_3_O_4_/ZnO composite and some previously reported photocatalysts.
