# Supplementary material for: Facile Preparation of Magnetically Separable Fe3O4/ZnO Nanocomposite with Enhanced Photocatalytic Activity for Degradation of Rhodamine B
Source: Nanomaterials (Basel). 2024 May 24;14(11):926. doi: 10.3390/nano14110926 (PMC11173383; doi:10.3390/nano14110926)
Supplement: Supplementary file 1 [file nanomaterials-14-00926-s001.zip › nanomaterials-3011177-supplementary.pdf]

Supplementary Materials

Facile Preparation of Magnetically Separable Fe_3O_4/ZnO Nanocomposite with Enhanced Photocatalytic Activity for Degradation of Rhodamine B

Li Qi [1], Siyu Wang [2], Yun Liu [1], Peng Zhao [2,3], Jing Tian [2], Baolin Zhu [2,3], Shoumin Zhang [2,3], Wenqi Xie [1] and Huanhuan Yu [1,2,*]

[1] College of Chemistry and Environmental Science, Shangrao Normal University, Shangrao 334001, China; seven820620@126.com (L.Q.); m18296897976@163.com (Y.L.); xwq2001@126.com (W.X.)

[2] College of Chemistry, Nankai University, Tianjin 300071, China; siyu2142021@163.com (S.W.); zhaopeng@nankai.edu.cn (P.Z.); tianjingnk@nankai.edu.cn (J.T.); zhubalin@nankai.edu.cn (B.Z.); zhangsm@nankai.edu.cn (S.Z.).

[3] The Key Laboratory of Advanced Energy Materials Chemistry (Ministry of Education), Nankai University, Tianjin 300071, China

* Correspondence: yuhuanhuan08@163.com; Tel.: +86-15620206206

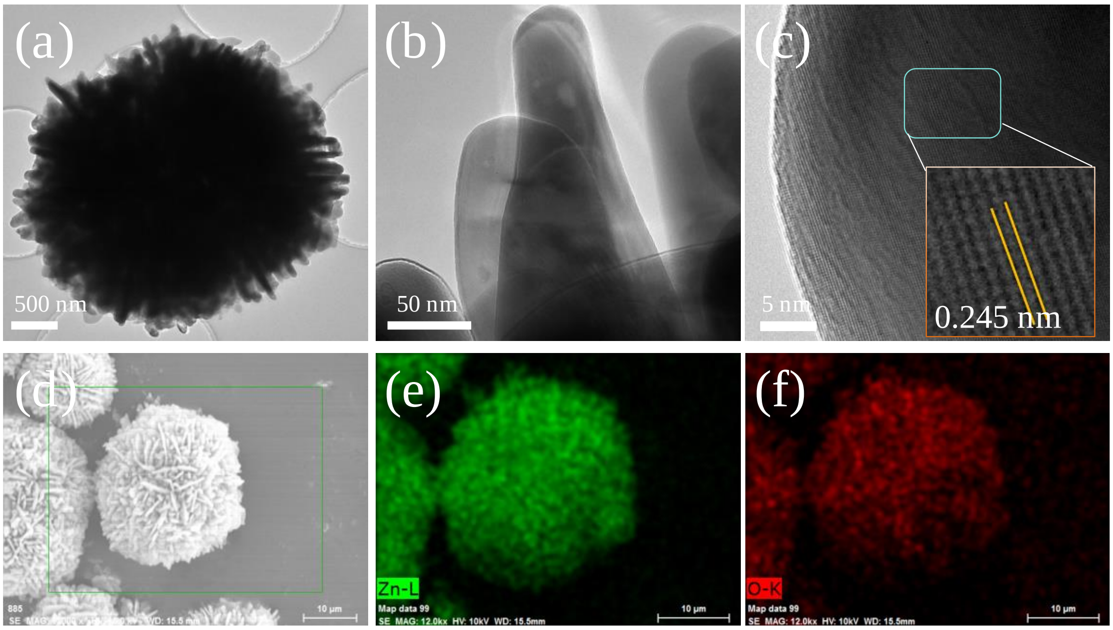

Figure S1 TEM image (a), HRTEM image (b), enlarged HRTEM image (c), and SEM elemental mapping for O, and Zn (d-f) of ZnO calcined at 500 °C.

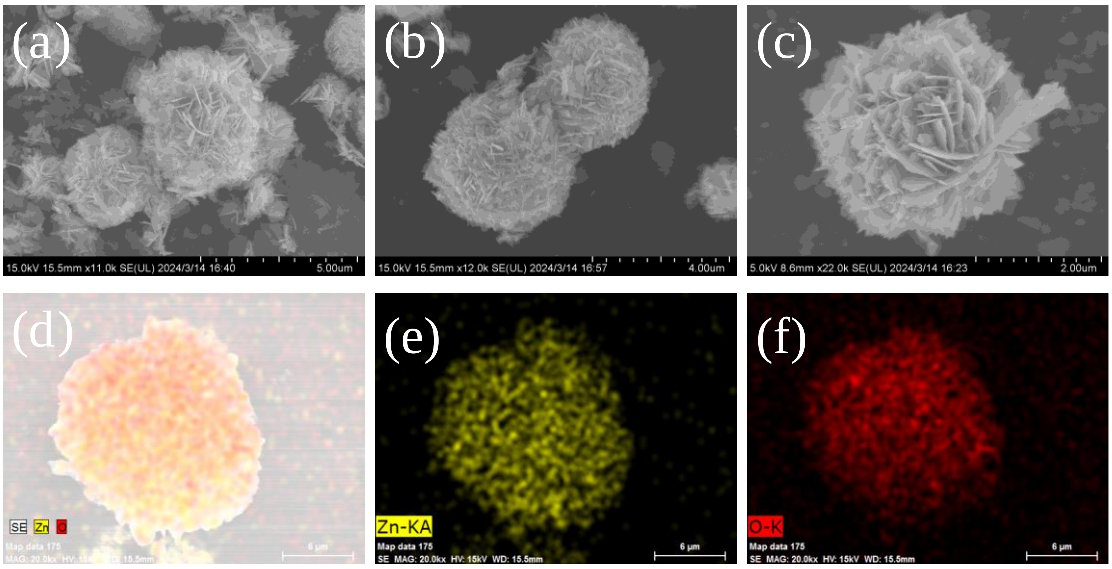

Figure S2 SEM images (a–c), elemental mapping for O and Zn (d–f) of ZnO dried at 80 °C.

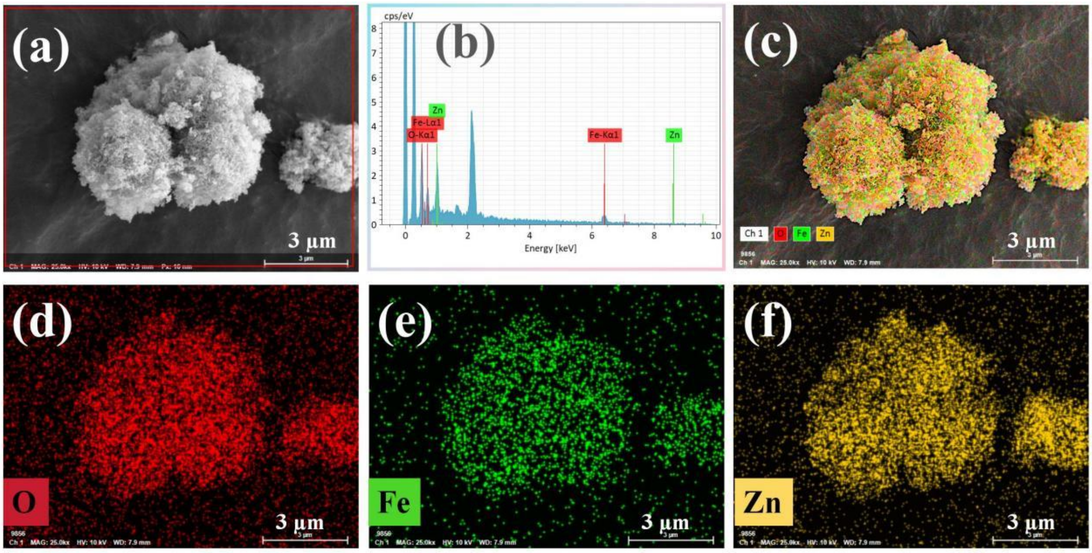

Figure S3 SEM image (a) with EDS image (b) and elemental mapping for O, Fe, and Zn (c-f) of Fe_3O_4/ZnO.

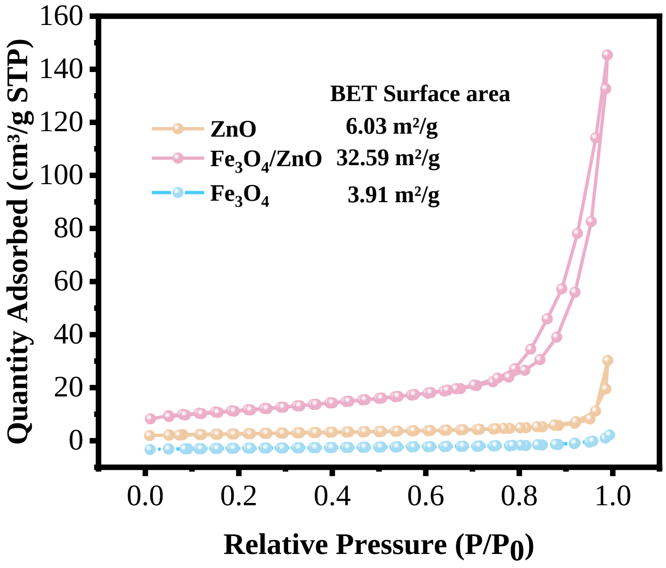

Figure S4 The N_2 adsorption-desorption isotherms of ZnO, Fe_3O_4/ZnO, and commercial Fe_3O_4.

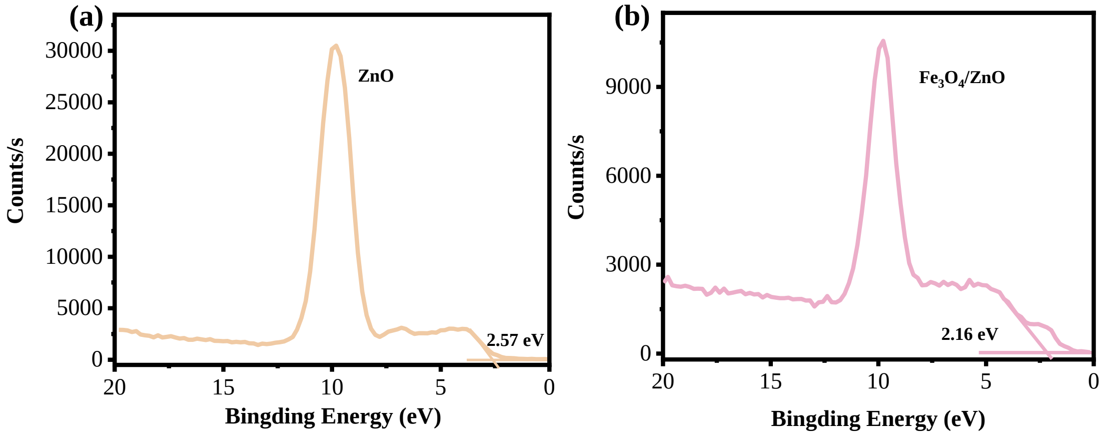

Figure S5 VB-XPS spectra of ZnO (a) and Fe_3O_4/ZnO (b).

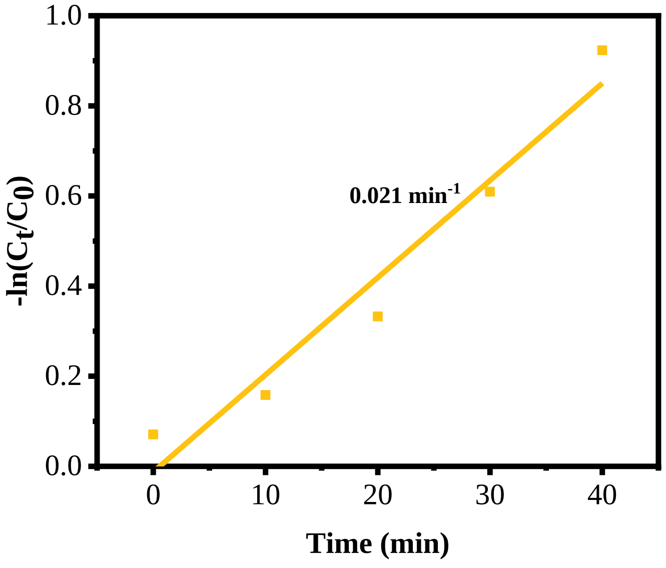

Figure S6 The corresponding first order kinetics constant for ZnO cataylst.

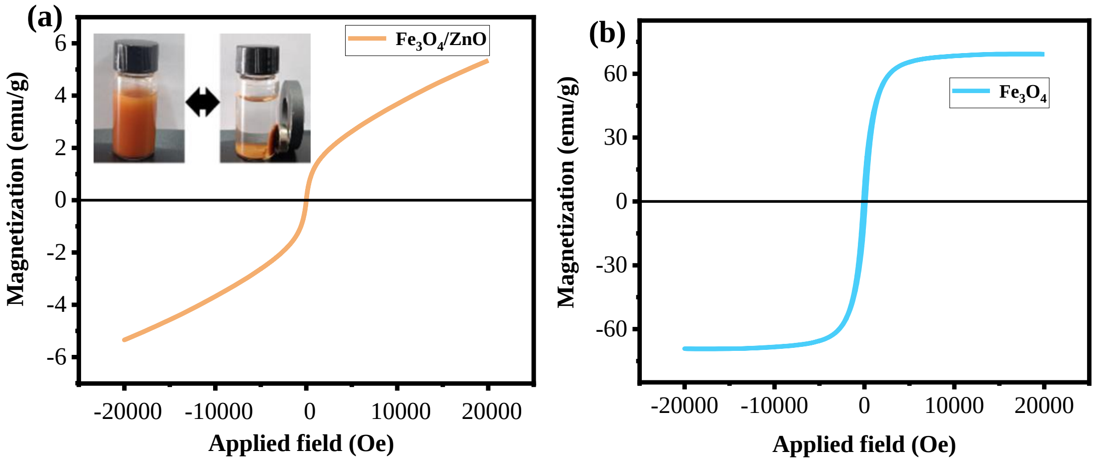

Figure S7 Magnetization curves for Fe_3O_4/ZnO nanocomposite (a) and commercial Fe_3O_4 nanoparticles. Inset figure in Fig. a shows the separation process of Fe_3O_4/ZnO from the degraded RhB solution by using a magnet.

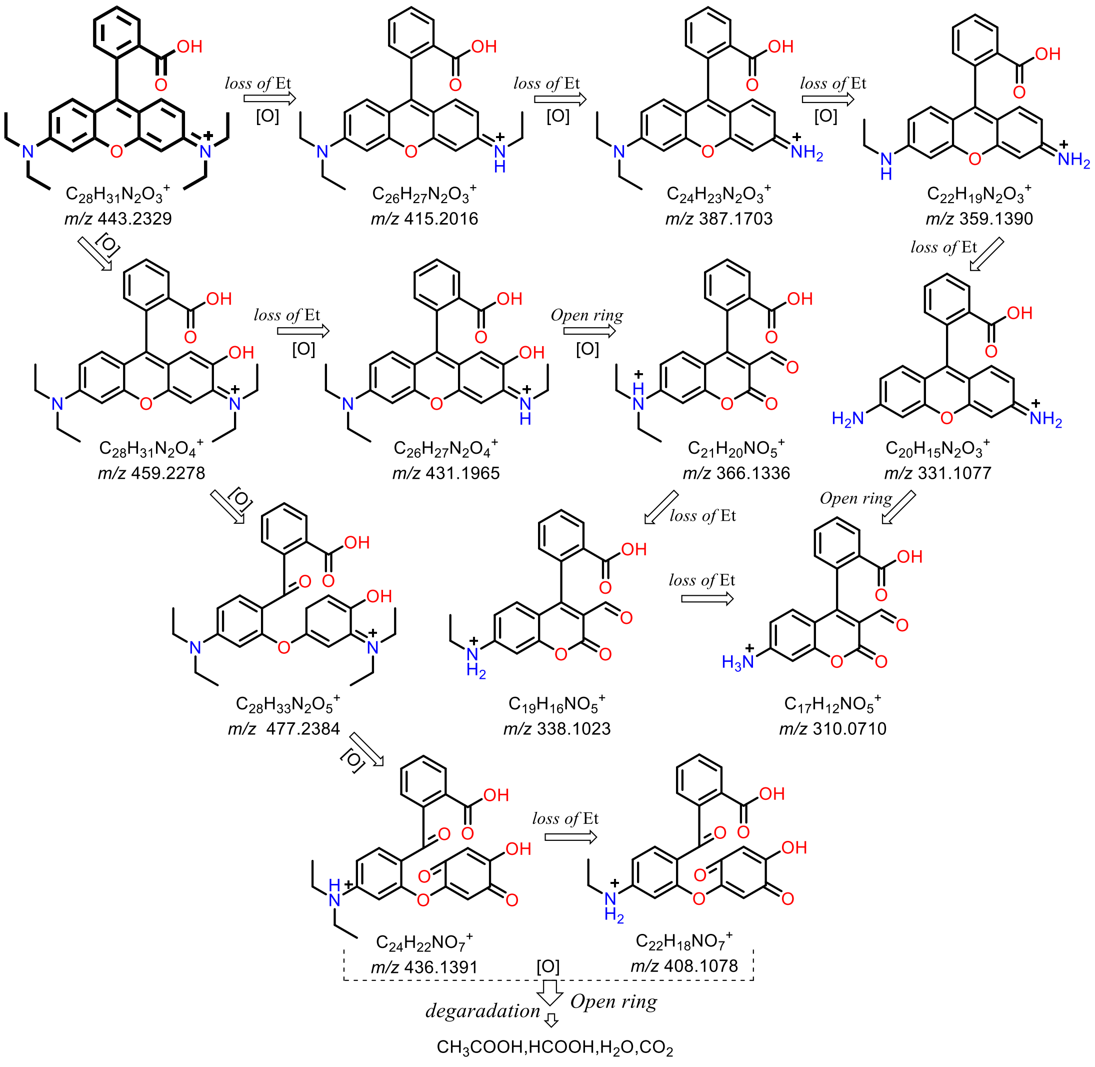

Figure S8 Possible degradation pathways of RhB.

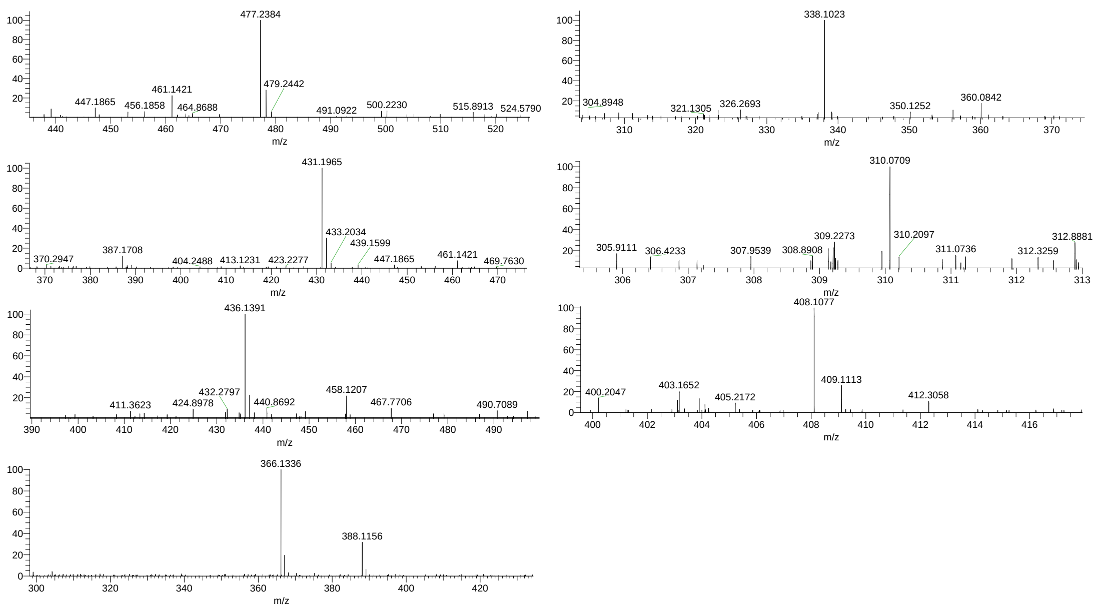

Figure S9 Mass spectra of main intermediates of RhB solutions after irradiation for 20 min under simulated UV light.

Table S1. Comparisons of RhB photocatalytic degradation between Fe_3O_4/ZnO composite and some previous reported photocatalysts.

Photocatalyst	Pollutant	k (min^{-1})	Light source	Reference
Zn3Ti1-LDO-6	RhB	0.04496	UV-light	[3]
Co, Fe-MIL88/MCC composite	RhB	0.07745	500 W halogen lamp	[5]
Ag@ZnO	RhB	0.025	UV-light	[6]
Fe-doped TiO_2/SnO_2 heterojunction	RhB	0.017	250 W Osram lamp	[15]
NiS/ZnO nanocomposite	RhB	0.0246	UV-light (254 nm)	[18]
ZnO–SnO_2 Ceramic Nanofibers	RhB	0.0166	UV-light	[26]
Fe_3O_4@ZnO@ZIF-8	RhB	0.0292	Visible light	[27]
Fe-doped g-C_3N_4	RhB	~0.057	300 W Xe lamp (λ≥420nm)	[29]
Fe_3O_4/ZnO	RhB	0.081	500 W halogen lamp	This work
